# Perceived exertion as a moderator of psychological changes during nature-based exercise among U.S. service members

**DOI:** 10.3389/fspor.2026.1754354

**Published:** 2026-04-20

**Authors:** Nicholas P. Otis, Hayley C. Myers, Lisa H. Glassman, Kim T. Kobayashi Elliott, Betty Michalewicz-Kragh, Kristen H. Walter

**Affiliations:** 1 Psychological Health & Readiness, Naval Health Research Center, San Diego, CA, United States; 2 Leidos, Inc., San Diego, CA, United States; 3 Department of Public Health, Naval Medical Center San Diego, San Diego, CA, United States

**Keywords:** blue space, dose-response, exercise outside, green exercise, major depressive disorder, military mental health, nature exposure, recreation therapy

## Abstract

**Introduction:**

Surf and hike therapies have been shown to significantly reduce depression symptom severity. However, the factors that moderate depression symptom reduction are not well understood and may support treatment recommendations. To identify potential moderators of symptom improvement, this study analyzed whether perceived exertion affected changes in depression symptom severity during surf or hike therapy.

**Methods:**

Participants were 86 active duty service members with major depressive disorder from a previous randomized clinical trial. Exertion was measured using the modified Borg Rating of Perceived Exertion Scale and assessed immediately after each of 6 weekly exercise therapy sessions. Study outcomes were depression/anxiety (4-item Patient Health Questionnaire-4) and positive affect (Positive Affect Schedule) scores from pre- to postsession.

**Results:**

Multilevel models demonstrated that surf and hike therapies significantly decreased depression/anxiety symptom severity and positive affect from pre- to postsession (*p*s < 0.001). Exploratory condition-specific analyses revealed that an average level of relative perceived exertion was associated with the greatest changes in depression/anxiety (*p* = 0.028) and positive affect (*p* = 0.074) during surf therapy. In hike therapy, higher and lower relative perceived exertion levels were related to greater reductions in depression/anxiety (*p* = 0.084), but exertion was not associated with changes in positive affect (*p* = 0.155).

**Discussion:**

Results suggest that the optimal level of perceived exertion required for maximizing benefit to depression/anxiety and positive affect may depend on the specific activity. Future investigations could consider examining other influences and contexts of exercise (e.g., environmental, social) to elucidate reasons for symptom change and maximize benefit for depression treatment.

## 1 Introduction

Major depressive disorder (MDD) is a significant public health burden associated with substantial societal and individual consequences. The prevalence of MDD is notably higher among U.S. service members than in the general population (1), making effective interventions a priority for both military and public health. Military-specific factors, such as deployment; frequent relocation; and structure, rules, and regulations, can influence the development and maintenance of MDD in service members (2). Fortunately, several evidence-based treatments effectively reduce MDD symptoms and are recommended for this population by the Department of Veterans Affairs (VA)/Department of Defense (DoD) Clinical Practice Guidelines for the Management of MDD (3). These treatments include psychotherapies such as acceptance and commitment therapy, behavioral activation, and cognitive behavioral therapy; and pharmacotherapies such as bupropion, vilazodone, and selective serotonin reuptake inhibitors (3). However, despite the evidence supporting these treatments, drawbacks may limit their benefits. Service members may not prefer evidence-based treatments, have access to them (4–6), or respond to them (7). In addition, they may require adjunctive care. Together, these findings highlight the need to explore additional treatment options that effectively ameliorate depression symptoms in this population.

Exercise is a widely recognized and effective strategy for managing and alleviating depression symptoms. In meta-analytic reviews, exercise has been shown to improve symptoms of MDD [e.g., (8–10)] with moderate effect sizes compared to usual care (i.e., an active control condition) (11). When integrated into treatment plans in a guided, progressive manner, exercise can be well-received and tolerated by individuals with MDD with minimal contraindications, less stigma, high satisfaction rates, and low rates of dropout and adverse events (12–15). Thus, exercise may alleviate common drawbacks of traditional depression treatments. Furthermore, a growing body of evidence suggests that exercising in natural or “green” environments offers additional psychological benefits compared to indoor or built environments (i.e., non-natural spaces) (16–18). Exercise outside has been shown to reduce depression symptoms (19–23), including among individuals with MDD (24). Although outdoor exercise is a promising treatment for depression, specific factors affecting symptom reduction remain underexplored (25). A better understanding of these underlying factors and processes could inform how outdoor exercise interventions are delivered as depression treatment (e.g., dosing).

One crucial parameter in exercise prescription—and a key factor influencing psychological responses to exercise—is intensity, commonly quantified through subjective Ratings of Perceived Exertion (RPE). RPE reflects an individual's perceived level of effort and fatigue during exercise (26). Although studies have demonstrated that both objective (27) and subjective (28) exercise intensity are predictors of mood and affect changes, the optimal “dose” of intensity remains a subject of debate (29). For example, in a recent meta-analysis of randomized clinical trials (RCTs) for MDD, results showed that depression symptoms improve linearly with exertion (29); however, another meta-analysis found that moderate intensity produced the greatest symptom reductions (e.g., a U shape) (8). There is also limited understanding of how perceived exertion interacts with the unique contextual factors of different exercise environments to influence mental health outcomes (29). The recognized psychological benefits of green exercise are often attributed to the combination of physical activity and exposure to nature (17, 30). Although the relationship between RPE and affect has been studied in indoor settings, the role of perceived exertion as a moderator in nature-based exercise has not been investigated, creating a gap in the literature. Consequently, it remains unclear whether the same level of perceived exertion elicits a similar psychological response across different outdoor activities (e.g., the complex, variable challenge of surfing vs. the consistent, self-paced effort of hiking). A deeper understanding of this relationship is essential for informing precision medicine approaches that maximize the therapeutic potential of nature-based exercise for individuals with MDD (30–33).

The current study aims to address this knowledge gap and improve dosing recommendations by examining whether perceived exertion moderates changes in depression and affect across two distinct outdoor activities. The investigation is a secondary analysis of data from an RCT that demonstrated the effectiveness of surf and hike therapies for active duty service members with MDD (24). The parent trial investigated changes in depression and related symptoms over time and within each session. Within-session results showed that both interventions produced significant reductions in depression/anxiety symptoms and increases in positive affect across six sessions. Building directly upon these results, the current study examined whether within-person, session-to-session fluctuations in RPE moderated the acute changes seen in depression/anxiety symptoms and positive affect within surf and hike therapy conditions. Based on the “sweet spot” theory of exercise-affect (28), we hypothesized a curvilinear (inverted-U/U-shaped) relationship between relative RPE and psychological outcomes. Specifically, we anticipated that the maximum acute benefit, defined as the greatest symptom reduction and affect increase, would occur at participants' average level of relative perceived exertion.

## 2 Methods

### 2.1 Participants

Participants in the parent study (24) consisted of 96 active duty service members referred to the Wounded, Ill, and Injured Wellness Department at Naval Medical Center San Diego (NMCSD) between January 2018 and March 2020. Participants were eligible if they met criteria for MDD based on the Diagnostic and Statistical Manual of Mental Disorders, Fifth Edition (DSM-5) (34), as assessed with the Mini International Neuropsychiatric Interview version 7.0 (MINI-7) (35). Service members who previously participated in the surf or hike therapy programs were excluded. Following the eligibility screening, service members were randomly assigned to either surf therapy (*n* = 48) or hike therapy (*n* = 48). All participants were medically cleared by a provider at NMCSD prior to beginning the program. Service members who attended at least one surf or hike therapy session (*N* = 86) were included in the current study.

### 2.2 Program

The surf and hike therapy programs were available as standard care options at NMCSD. Each program was delivered once per week for 6 weeks, with each session lasting 3–4 h. Sessions followed a cohort-based format, with groups consisting of approximately 20 service members. No formal psychotherapeutic techniques were administered during the sessions; rather, the activities themselves (i.e., surfing, hiking) served as the therapeutic component, consistent with prior literature (36). Sessions took place on public beaches and trails throughout San Diego County.

### 2.3 Procedure

Service members completed a series of assessments at preprogram, postprogram, and 3-month follow-up. For the purposes of the current analysis, preprogram measures were used to characterize the sample. To capture symptom changes during exercise sessions, participants completed self-report assessments before and after each session. Further details about the parent study, which compared the psychological outcomes of water-based therapy to a land-based active control, have been previously published (24). The NMCSD Institutional Review Board approved study protocol, and all participants provided written informed consent.

### 2.4 Measures

The modified Borg Rating of Perceived Exertion Scale (Borg RPE) (26) was used to assess participants' overall perceived exertion at the conclusion of each exercise session. This self-report measure is an author-developed modification of the original 6–20 Borg RPE scale, rated from 0 (*nothing at all*) to 10 (*very, very hard*) with a 0.5 option between 0 and 1 (see Supplementary material 1) (26). The modified Borg RPE (sometimes referred to as the CR10) is often used for ease of interpretation among clinical populations and maintains excellent repeated-measures reliability and correlation with the original (37). Consistent with scale instructions, participants were instructed to rate their overall feeling of effort and exertion, taking into account factors such as breathing, heart rate, and muscle fatigue during the session.

Depression/anxiety and positive affect were evaluated before and after each exercise session using the 4-item Patient Health Questionnaire (PHQ-4) and Positive Affect Schedule (PAS), respectively. The PHQ-4 (38) is a popular, low-burden self-report screening questionnaire that assesses symptom severity via two depression items from the 9-item Patient Health Questionnaire (PHQ-9) (39) and two anxiety items from the 7-item Generalized Anxiety Disorder Scale (GAD-7) (40). The PHQ-4 was selected by the parent study (24) for both its brevity and clinical relevance for MDD. Items on the PHQ-4 are scored from 0 (*not at all*) to 3 (*nearly every day*) and then summed, with higher total scores reflecting greater depression and anxiety symptom severity. The PHQ-4 items typically assess symptoms over the “past week”; however, the current study assessed symptoms “at this moment” to evaluate changes following each session. This modification is supported by research demonstrating the PHQ-4's sensitivity to symptom changes within 4- to 8-hour periods (22, 23). Furthermore, variation in recall periods (e.g., “past week” vs. “now”) have not been shown to differentially affect mental health symptom reporting (41, 42). The sample's reliability was *α* = 0.76–0.88 across sessions.

The Positive Affect Schedule (PAS), a subscale of the Positive and Negative Affect Schedule (PANAS) (43), measured participants' positive emotional states before and after each exercise session. This self-report instrument consists of 10 adjectives (e.g., “interested,” “enthusiastic,” “alert”) that describe positive affect “in the past few hours.” Participants rated these positive emotions on a 5-point Likert scale ranging from 1 (*very slightly or not at all*) to 5 (*extremely*). Higher scores on the PAS indicate greater positive affect. Internal consistency in this sample ranged from *α* = 0.91–0.98 across sessions.

At the preprogram assessment, participants provided demographic and concurrent depression treatment information. Depression symptom severity was evaluated with the clinician-rated Montgomery–Åsberg Depression Rating Scale (MADRS) (44) and the self-reported PHQ-9 (39). Additionally, a Fitbit Charge 2 device (consumer accelerometer) was given to participants to track physical activity over the course of the study. Physical activity levels during the study were quantified in metabolic equivalent minutes (MET mins) (45) and used to describe the sample.

### 2.5 Data analysis

To examine descriptive differences between surf and hike therapy groups, *t*-tests and chi-square tests were used for continuous and categorical variables, respectively. Multilevel modeling (MLM) determined the influence of perceived exertion on depression/anxiety symptoms and positive affect over the course of a single exercise session. MLMs used a step-up model-building process; logical covariance matrices and variables were selected based on model fit according to the Akaike Information Criterion with respect to the number of parameters specified. Based on null findings from previous work (24, 46) and Akaike Information Criterion in initial models, exercise condition and concurrent depression treatment variables were not used as fixed effects in final models. In addition, because our research question focused on whether *person-relative fluctuations in perceived exertion* were associated with changes in outcomes, we modeled only within-person Borg scores (person-mean centered) and their quadratic term. Between-person Borg scores were not included, as these reflect stable reporting tendencies rather than session-level variation. Excluding them also reduced model complexity, improving interpretability without affecting primary conclusions. Lastly, both the linear and quadratic within-person terms were included based on conflicting literature as to whether a linear or U-shaped relationship exists between exertion and psychological improvements (8, 29).

Final MLMs were structured such that random effects included intercept, time, session, and the crossed effect of time × session, all set as an effect of participant with a first-order autoregressive covariance matrix. Repeated effects included the crossed effect of time × session as an effect of participant with a compound symmetry covariance matrix. Fixed effects consisted of time (pre- to postsession), person-mean centered Borg RPE rating, person-mean centered Borg RPE rating^2^, as well as time × Borg RPE rating, and time × Borg RPE rating^2^. Separate models were run for PHQ-4 and PAS outcomes. All final models used restricted maximum likelihood to account for missing data. Analyses were conducted using IBM SPSS (Version 29.0.0).

## 3 Results

Sample characteristics can be found in Table 1. At preprogram, the overall sample reported *moderate* depression symptom severity via clinician-assessed MADRS scores (*M* = 27.0, *SD* = 8.2) and *moderately severe* depression symptom severity via self-reported PHQ-9 scores (*M* = 17.0, *SD* = 4.8). Ninety-one percent (*n* = 78) of service members met or exceeded the U.S. Physical Activity Guidelines (≥450 MET mins/week) (47) from pre- to postprogram. The average number of MET minutes per worn week from pre- to postprogram was 977 (*SD* = 440). On average, service members completed 4.2 (SD = 1.3) out of six possible exercise therapy sessions. Service members randomized to hike therapy reported significantly higher preprogram PHQ-9 scores (*MD* = 2.4, SE = 1.0, *p* = 0.022) compared to surf therapy, although this difference was not clinically significant (48). Additionally, those in hike therapy attended approximately one session more than those in surf therapy (*MD* = 0.7, SE = 0.3, *p* = 0.010). Exercise therapy groups did not differ on any other preprogram characteristics.

**Table 1:** Sample characteristics.

Characteristic	Total sample *N* = 86	Surf therapy *n* = 44	Hike therapy *n* = 42
Age, years, *M* (*SD*)	28.3 (5.8)	29.3 (6.4)	27.2 (5.0)
Sex, *n* (%)			
Female	45 (52.3)	21 (47.7)	24 (57.1)
Male	41 (47.7)	23 (52.3)	18 (42.9)
Race/ethnicity, *n* (%)			
Asian or Asian-American	3 (3.5)	3 (6.8)	0 (0.0)
Black or African American	13 (15.1)	6 (13.6)	7 (16.7)
Hispanic, Latino, or Spanish origin	15 (17.4)	7 (15.9)	8 (19.0)
Multiracial	18 (20.9)	9 (20.5)	9 (21.4)
Native American or Alaska Native	1 (1.2)	0 (0.0)	1 (2.4)
White	36 (41.9)	19 (43.2)	17 (40.5)
Rank, *n* (%)			
E1–E4	32 (37.2)	13 (29.5)	19 (45.2)
E5–E9	51 (59.3)	29 (65.9)	22 (52.4)
Officer	3 (3.3)	2 (4.5)	1 (2.4)
Concurrent pharmacotherapy for depression, *n* (%)	59 (68.6)	31 (70.5)	28 (66.7)
Sessions attended, *M* (*SD*)^a^^,^^b^	4.2 (1.3)	3.9 (1.3)*	4.6 (1.1)*
Completion of assigned program, *n* (%)^b^^,^^c^	68 (82.9)	37 (84.1)	31 (81.6)
Physical activity (MET mins/week), *M (SD)*^d^	977 (440)	1,034 (471)	917 (402)
Depression severity, *M* (*SD*)			
MADRS	27.0 (8.2)	25.8 (7.8)	28.2 (8.4)
PHQ-9	17.0 (4.8)	15.8 (4.7)*	18.2 (4.6)*

E, enlisted rank; MADRS, Montgomery–Åsberg Depression Ranking Scale; PHQ-9, 9-item Patient Health Questionnaire.

^a^ For fidelity, only sessions where the assigned modality was conducted are included. Occasionally, due to adverse weather, sessions consisted of alternative activities (e.g., visit to the National Surf Museum in Surf Therapy, strength and conditioning class in Hiking Therapy).

^b^ Because programs were halted due to the sudden onset of COVID-19, participants (*n* = 8) in the affected cohort were not counted in completion or attendance statistics.

^c^ Program completion was defined by the Naval Medical Center San Diego as missing no more than two sessions of the assigned modality.

^d^ Pre- to postprogram.

* *p* < 0.05.

Table 2 presents mean Borg RPE scores across sessions by exercise therapy type. The surf therapy group reported significantly higher levels of perceived exertion than the hike therapy group in Session 1 (MD = 2.7, SE = 0.6, *p* < 0.001) and Session 2 (MD = 2.0, SE = 0.6, *p* = 0.002). No significant differences in Borg RPE were found between conditions for Sessions 3–6 (*p*s > 0.109).

**Table 2:** Means and standard deviations of participants’ modified Borg RPE scale by exercise session and condition.

Condition	Session 1	Session 2	Session 3	Session 4	Session 5	Session 6
Surf	5.7 (2.7)	6.0 (2.7)	5.6 (2.9)	5.7 (3.1)	5.1 (3.5)	5.1 (3.3)
Hike	3.0 (2.3)	4.0 (2.3)	4.7 (2.1)	5.0 (2.4)	4.4 (2.5)	3.9 (2.7)

Borg RPE, modified Borg Rating of Perceived Exertion.

Means are reported outside the parentheses; standard deviations are reported in parentheses. The modified Borg RPE scale ranges from 0 to 10 and includes a 0.5 between 0 and 1.

Between-condition *p*-values were as follows: Session 1 *p* < 0.001, Session 2 *p* = 0.002, Session 3 *p* = 0.109, Session 4 *p* = 0.329, Session 5 *p* = 0.397, Session 6 *p* = 0.376.

In multilevel models, initial models without covariates indicated significant symptom change following an exercise session for both outcomes. That is, across participants, depression/anxiety symptoms significantly decreased [*B* = − 2.99, 95% CI (−3.30, −2.68), *p* < 0.001] and positive affect increased [*B* = 7.79, 95% CI (6.77, 8.81), *p* < 0.001]. However, in the final models, interactions between time and within-person perceived exertion (linear or quadratic) were not significant for either the PHQ-4 (*p*s = 0.828,.459) or PAS (*p*s = 0.991,.482). These findings showed that fluctuations in exertion did not modify trajectories of depression/anxiety or affect in the overall sample. Table 3 contains these full sample estimates of fixed effects for final multilevel models.

**Table 3:** Fixed effects for final multilevel models on perceived exertion and acute symptom change (full sample).

Outcome	B (SE)	95% CI	*p*
PHQ-4			
Intercept	6.13 (0.25)	[5.63, 6.63]	<0.001
Time (pre- to postsession)	−3.19 (0.19)	[−3.56, −2.82]	<0.001
Borg RPE	0.06 (0.07)	[−0.07, 0.20]	0.371
Borg RPE × Time	0.02 (0.09)	[−0.17, 0.21]	0.828
Borg RPE^2^	0.01 (0.03)	[−0.05, 0.06]	0.850
Borg RPE^2^ × Time	0.03 (0.04)	[−0.04, 0.10]	0.459
PAS			
Intercept	24.11 (0.87)	[22.39, 25.83]	<0.001
Time (pre- to postsession)	8.55 (0.61)	[7.36, 9.74]	<0.001
Borg RPE	−0.30 (0.20)	[−0.70, 0.10]	0.141
Borg RPE × Time	0.00 (0.28)	[−0.55, 0.55]	0.991
Borg RPE^2^	−0.07 (0.08)	[−0.23, 0.09]	0.408
Borg RPE^2^ × Time	−0.08 (0.11)	[−0.29, 0.14]	0.484

Borg RPE, modified Borg Rating of Perceived Exertion; PAS, Positive Affect Schedule; PHQ-4, 4-item Patient Health Questionnaire.

**Figure 1:**
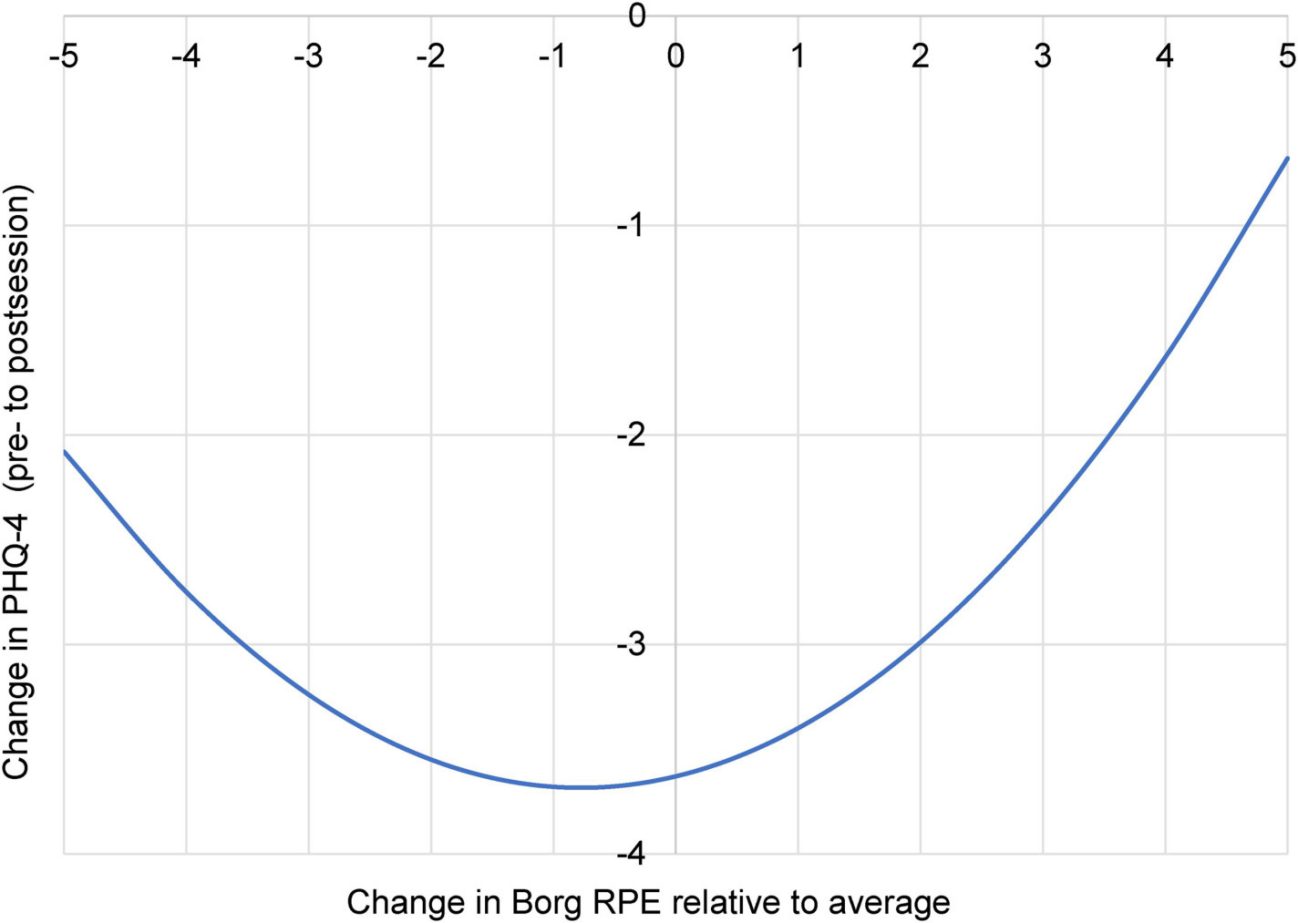
Depression/anxiety symptom change as related to relative perceived exertion during a surf therapy session. Borg RPE, modified Borg Rating of Perceived Exertion scale; PHQ-4, 4-item Patient Health Questionnaire. More negative change on the *Y*-axis indicates a greater reduction in symptoms. More positive or negative exertion on the *X*-axis indicates a higher or lower perceived exertion, respectively, relative to one's average across all sessions. Time × Borg RPE^2^ interaction *p* = 0.028, *B* = 0.09.

**Figure 2:**
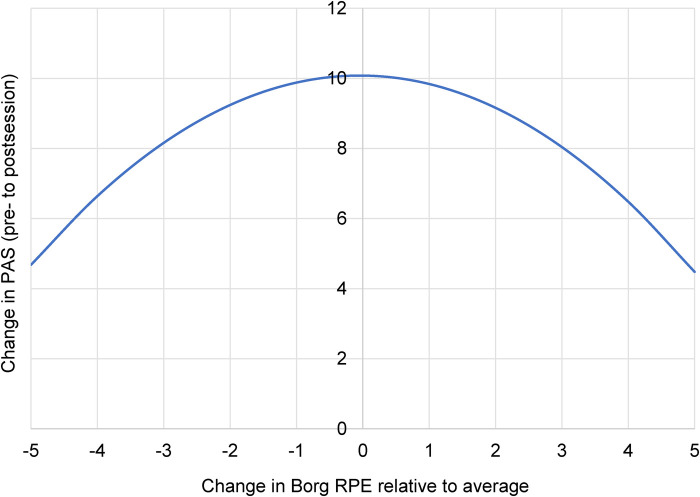
Positive affect change as related to relative perceived exertion during a surf therapy session. Borg RPE, modified Borg Rating of Perceived Exertion scale; PAS, Positive Affect Schedule. More positive change on the *Y*-axis may indicate a greater increase in positive affect. More positive or negative exertion on the *X*-axis may indicate a higher or lower perceived exertion, respectively, relative to one's average across all sessions. Time × Borg RPE^2^ interaction *p* = 0.074, *B* = − 0.22.

Condition-specific models suggested modest, activity-dependent effects (Table 4). Due to the reduced power of these subgroup analyses, these findings should be interpreted with caution and as hypothesis-generating rather than confirmatory. In surf therapy, there was a significant quadratic interaction between time and within-person exertion on PHQ-4 scores [*B* = 0.093, 95% CI (0.010, 0.175), *p* = 0.028], such that higher or lower relative perceived exertion was associated with less reduction in depression/anxiety symptoms. Figure 1 depicts the regression equation. This figure illustrates an unbalanced “U” shape, where lower relative exertion results in less depression/anxiety improvement, and higher relative exertion results in *even less* depression/anxiety improvement. However, as depicted in Figure 1, all plotted values are in the negative part of the *Y*-axis, implying that although there is reduced change in depression/anxiety at these extremes of relative exertion, the change is still likely to remain beneficial. A marginal quadratic effect also emerged for PAS in surf therapy [*B* = − 0.224, 95% CI (−0.470, 0.022), *p* = 0.074], suggesting that improvements in positive affect may diminish at very high or very low levels of relative exertion. Like Figure 1, Figure 2 still depicts *overall improvements* in positive affect at these extreme ends of relative exertion, even if attenuated in this marginal effect. In sum, it appears that average relative perceived exertion is associated with the greatest changes in depression/anxiety symptoms and positive affect during surf therapy.

**Table 4:** Fixed effects for final multilevel models on perceived exertion and acute symptom change (by exercise condition).

Outcome	Surf therapy			Hike therapy		
	B (SE)	95% CI	*p*	B (SE)	95% CI	*p*
PHQ-4						
Intercept	5.82 (0.32)	[5.17, 6.46]	<0.001	6.64 (0.38)	[5.89, 7.39]	<0.001
Time (pre- to postsession)	−3.63 (0.27)	[−4.16, −3.10]	<0.001	−2.64 (0.26)	[−3.16, −2.11]	<0.001
Borg RPE	−0.10 (0.10)	[−0.28, 0.09]	0.321	0.22 (0.10)	[0.03, 0.41]	0.026
Borg RPE × Time	0.14 (0.13)	[−0.12, 0.40]	0.301	−0.02 (0.13)	[−0.28, 0.24]	0.861
Borg RPE^2^	−0.01 (0.03)	[−0.07, 0.05]	0.714	0.01 (0.05)	[−0.09, 0.11]	0.833
Borg RPE^2^ × Time	0.09 (0.04)	[0.01, 0.18]	0.028	−0.10 (0.06)	[−0.22, 0.01]	0.084
PAS						
Intercept	26.29 (1.17)	[23.94, 28.64]	<0.001	21.68 (1.11)	[19.46, 23.89]	<0.001
Time (pre- to postsession)	10.08 (0.86)	[8.38, 11.77]	<0.001	6.48 (0.88)	[4.75, 8.21]	<0.001
Borg RPE	−0.35 (0.28)	[−0.91, 0.20]	0.214	−0.32 (0.29)	[−0.90, 0.25]	0.271
Borg RPE × Time	−0.02 (0.38)	[−0.77, 0.74]	0.965	−0.14 (0.40)	[−0.93, 0.65]	0.727
Borg RPE^2^	−0.07 (0.09)	[−0.25, 0.12]	0.491	0.02 (0.16)	[−0.29, 0.33]	0.909
Borg RPE^2^ × Time	−0.22 (0.12)	[−0.47, 0.02]	0.074	0.27 (0.19)	[−0.10, 0.65]	0.155

Borg RPE, modified Borg Rating of Perceived Exertion; PAS, Positive Affect Schedule; PHQ-4, 4-item Patient Health Questionnaire.

**Figure 3:**
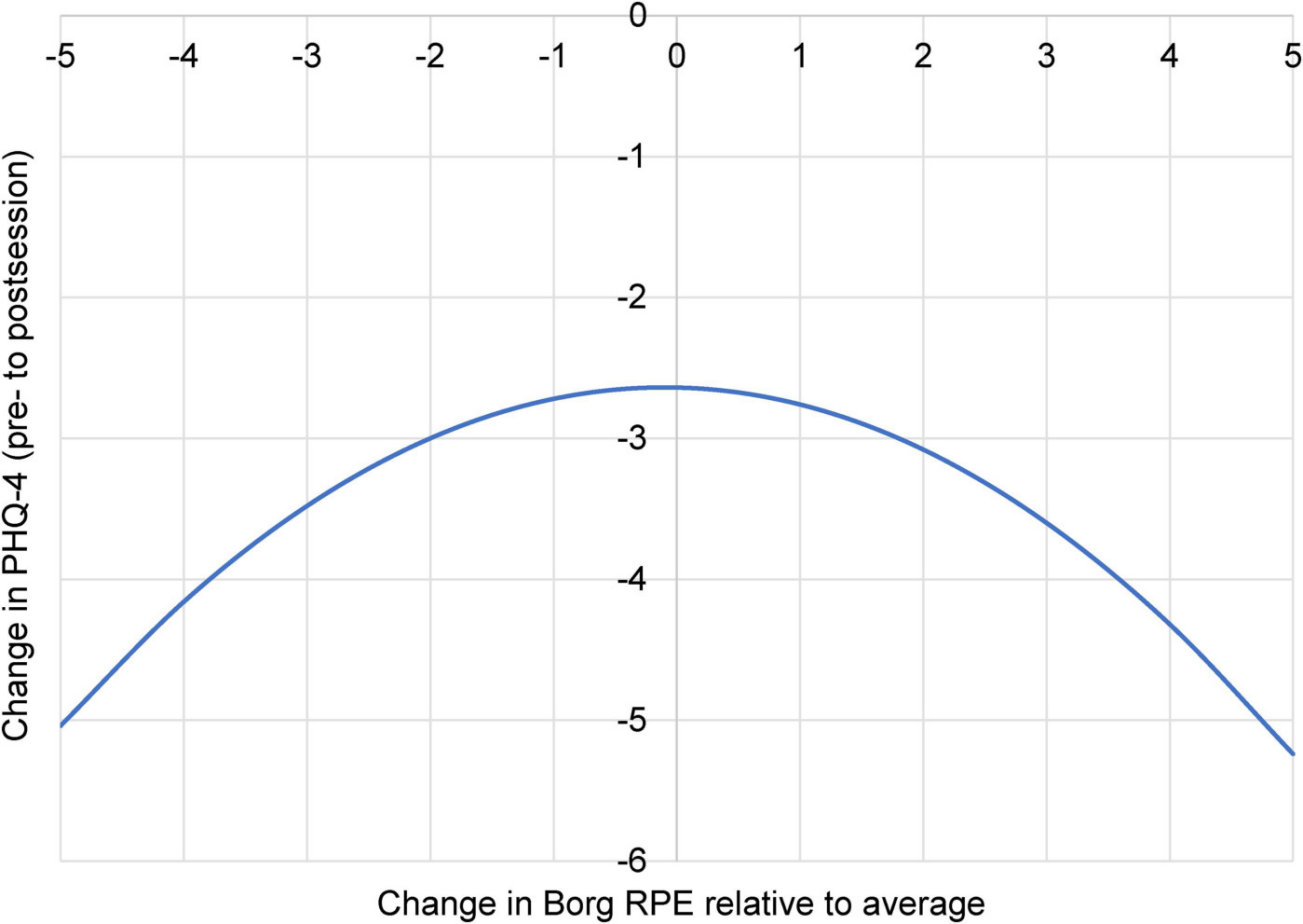
Depression/anxiety symptom change as related to relative perceived exertion during a hike therapy session. Borg RPE, modified Borg Rating of Perceived Exertion scale; PHQ-4, 4-item Patient Health Questionnaire. More negative change on the *Y*-axis may indicate a greater reduction in symptoms. More positive or negative exertion on the *X*-axis may indicate a higher or lower perceived exertion, respectively, relative to one's average across all sessions. Time × Borg RPE^2^ interaction *p* = 0.084, *B* = − 0.10.

For hike therapy, a marginal quadratic interaction effect emerged for the PHQ-4 [*B* = − 0.104, 95% CI (−0.222, 0.014), *p* = 0.084], suggesting that—contrary to surf therapy—a *higher or lower* relative perceived exertion may be linked with *greater* reductions in depression/anxiety symptoms. Figure 3 depicts this marginally significant “inverted U” pattern, whereby *the greatest* improvement in depression/anxiety symptoms was associated with *very low- or very high levels* of relative exertion. Positive affect, however, did not show this pattern. Although positive affect significantly improved over time, no significant interaction effects emerged between time and Borg ratings, indicating that relative exertion did not moderate affect in this activity. Taken together, higher and lower relative perceived exertion levels may relate to greater reductions in depression/anxiety while hiking, but not positive affect.

## 4 Discussion

Research demonstrates that outdoor exercise interventions can effectively reduce depression symptoms; however, less is known about the factors and processes driving this symptom change. The current study aimed to examine within-session effects of perceived exertion levels on psychological outcomes during two distinct forms of nature-based therapy: surf and hike therapies. Consistent with prior research on the benefits of exercise and nature exposure, both therapies were associated with significant acute symptom reductions and increases in positive affect in the overall sample. However, the most compelling findings emerged from the condition-specific analyses, which revealed that the optimal “dose” of perceived exertion varied by activity type.

Our results suggest a curvilinear relationship between perceived exertion and therapeutic benefit, with patterns differing by modality. In surf therapy, the greatest improvements in both depression/anxiety and positive affect were associated with an individual's *average* relative perceived exertion. Specifically, the relationship between exertion and depression/anxiety followed a U-shaped pattern where the greatest symptom reductions occurred at average relative effort. This signifies that while all levels of exertion were beneficial, improvements were attenuated at the extremes of relative perceived exertion. Of further note, this U shape was unbalanced, like a soft “check mark” (Figure 1). This indicates that while lower relative exertion results in less depression/anxiety improvement relative to average, higher relative exertion results in *even less* depression/anxiety improvement (closer to zero). This may be explained by variability in surfing conditions, where low exertion may indicate few waves and lots of “floating time” on the surfboard, resulting in lower levels of exercise engagement. In contrast, high exertion may denote surf conditions beyond one's ability, possibly creating anxiety, fear, frustration, or other distressful states. This optimal range of exertion extended to positive affect as well; an *inverted* U-shaped curve emerged, indicating that the most significant gains occurred at average relative exertion, while the extremes of perceived exertion resulted in the smallest increases (Figure 2).

For both outcomes, the greatest gains were achieved when participants felt they were working at their typical level of effort during a surf session. These findings align with literature on the “sweet spot” for exercise, suggesting that a sense of optimal challenge—which avoids the strain of overexertion and the boredom of under-exertion—is key to maximizing therapeutic benefits (49–51). This may be particularly relevant to surf therapy, where achieving a “flow state” is often cited as a core mechanism of change in mood, and a feeling of optimal challenge is a prerequisite for flow (52). However, because this study did not directly measure flow or skill-challenge balance, such interpretation remains speculative.

In contrast, the results for hike therapy suggested a different optimal dose of perceived exertion. For depression/anxiety symptoms, findings indicated a marginally significant U-shaped relationship, where the greatest symptom reduction was likely associated with the *extremes* of perceived effort. The least benefit—although still positive—was observed at their average or typical level of exertion. This finding is novel and contradicts the traditional inverted-U hypothesis for indoor exercise (28), suggesting that different mechanisms may be driving symptom reduction during hike therapy. For instance, an easy hike may facilitate a state of contemplative relaxation and mindfulness, while a difficult hike may provide a sense of mastery and self-efficacy, both of which can be therapeutic (51, 53). Future research should empirically test whether these processes are at play. Unlike surf therapy, positive affect in hiking was not moderated by perceived exertion, demonstrating that while hiking reliably increases positive affect, the extent of this increase is not dependent on how hard a person feels they are working.

Our finding that changes in positive affect were related to exertion in surf therapy but not hike therapy highlight potential avenues to explore in future research. It is possible that these activities may engage distinct affective pathways. Affect regulation research (54) identifies two primary systems in outdoor settings: drive (high arousal, challenge-oriented) and contentment (low arousal, restorative). For military populations, often characterized by a sense of purpose and mission focus, the vigorous and skill-demanding nature of surfing may predominantly activate drive-related affect. It is also possible that structural differences between the surf and hike therapies contribute to these differential effects: surf therapy was delivered 1:1 with an instructor, whereas hike therapy was largely autonomous. While self-selected exercise intensity is associated with increased pleasure (28), greater autonomy may be *less* beneficial for individuals with depression, possibly because low self-efficacy interferes with setting an appropriate level of challenge (55). The possibility that surf therapy may engage distinct affective pathways compared to hike therapy raises an interesting question to examine in future research efforts.

Results have important implications for clinicians, recreation therapists, and program managers in the design and delivery of nature-based therapies. First, they highlight the importance of individualizing the therapeutic experience based on the activity type. For surf therapy programs, focus should be placed on helping participants find their optimal level of challenge: one that feels “just right” and avoids extremes. Surf therapy instructors could guide participants to self-regulate their effort, encouraging them to find a sense of flow for the conditions encountered rather than focusing on extreme physical performance. Conversely, for hike therapy, staff might consider offering trail options with a range of difficulty levels for a given hike. Hike therapy instructors could tentatively recommend participants to either challenge themselves physically on more vigorous trails or engage in a low-effort, leisurely walk, as both appear to be more beneficial than a moderate-difficulty hike. The consistent finding across both conditions that perceived exertion is a significant moderator underscores the importance of discussing a person's perceived effort and challenge during a session.

Study results should be interpreted in the context of their limitations. First, the moderating effects of perceived exertion on depression-related outcomes are modest, with relatively small beta coefficients and marginal significance for some quadtratic terms. Consequently, the clinical magnitude of these effects may be limited, and results should be interpreted with caution. Statistical power may also be limited, particularly for condition-specific analyses that involved a quadratic pattern, which may have hindered our ability to detect more robust effects. These patterns warrant replication in larger samples. Second, the generalizability of these findings is restricted. A significant proportion of participants were already adequately physically active (91%), likely related to their occupation as service members. Results may not generalize to more sedentary populations with MDD or civilians. Third, data for exertion and psychological outcomes were derived from self-report measures that may be subject to recall or social desirability biases. It should also be noted that Borg RPE scores significantly differed between surf and hike therapies during the first two sessions. This may have been due to differential learning curves. While hike therapy participants completed “easy”, introductory trails during the first two weeks, surf therapy participants were first required to master novel physical skills (e.g., balancing and standing on a board). These differences may add a layer of complexity to early perceived exertion between therapies. Finally, the study did not examine factors beyond exertion, such as social connection or skill mastery, which may also influence psychological outcomes (51).

This study also has numerous strengths that extend the understanding of nature-based therapies for MDD. By examining perceived exertion as a moderator of psychological changes, this work identifies potential factors that drive symptom improvement during outdoor exercises. Additionally, the parent trial included both surf and hike therapies, lending a broader perspective on nature-based interventions. Analyses were conducted using multilevel modeling, a robust approach that is well-suited to within-person change; missing data; and longitudinal, repeated-measures designs. Although data for this study were largely derived from self-report measures, they are well-validated and commonly used in exercise psychology and treatment research, and among military populations. Furthermore, this study used clinician-administered diagnostic assessments and accelerometer data to determine participant eligibility and characterize the sample. All participants met diagnostic criteria for MDD, allowing outcomes to be evaluated within a clinical population for whom exercise may be particularly beneficial (51). Further, the trial was conducted in a real-world Military Health System setting, improving external validity.

## 5 Conclusion

In summary, both surf and hike therapies were associated with improvements in acute depression/anxiety symptoms and positive affect. However, study findings reveal a nuanced and activity-dependent relationship between perceived exertion and psychological outcomes. For surf therapy, the optimal perceived dose may be at an individual's average level of effort. In contrast, for hike therapy, the greatest benefits are linked to sessions that are perceived as either very easy or very hard compared to one's average. These results suggest that clinicians should move away from uniform intensity prescriptions and instead help patients find an activity-specific level of challenge that maximizes individual therapeutic benefit. While these results are exploratory, they provide a foundational framework for future research to empirically test mediators across different nature-based modalities, ultimately supporting the use of outdoor exercise as precision medicine for mental health care.

## Data Availability

The datasets generated and/or analyzed during the current study are not publicly available due to security protocols and privacy regulations, but they may be made available on reasonable request by the Naval Medical Center San Diego Institutional Review Board (contact phone +1.619.553.8400).

## Author disclaimer

KW, KKE, and BM-K are employees of the US Government. This work was prepared as part of their official duties. Title 17, USC §105 provides that copyright protection under this title is not available for any work of the US Government. Title 17, USC §101 defines a US Government work as work prepared by a military service member or employee of the US Government as part of that person's official duties. Report No. 25-100 was supported by the US Navy Bureau of Medicine and Surgery under work unit no. N1600. The views expressed in this article are those of the authors and do not necessarily reflect the official policy or position of the Department of the Navy, Department of Defense, nor the US Government. The study protocol was approved by the Naval Medical Center San Diego Institutional Review Board in compliance with all applicable federal regulations governing the protection of human subjects. Research data were derived from approved Naval Medical Center San Diego Institutional Review Board protocol number NMCSD.2017.0007.
