# Supplementary material for: Perceived exertion as a moderator of psychological changes during nature-based exercise among U.S. service members
Source: Front Sports Act Living. 2026 Apr 20;8:1754354. doi: 10.3389/fspor.2026.1754354 (PMC13137058; doi:10.3389/fspor.2026.1754354)
Supplement: Supplementary file 1 [file Supplementaryfile1.docx]

**Supplemental Material.**

**S1 The Modified Borg Rating of Perceived Exertion Scale**

Choose a number below from 0 to 10 that best describes your level of exertion **during today’s session**—0 means “no exertion at all” and 10 means “very, very hard.” Do not concern yourself with any one factor, such as leg pain or shortness of breath, but try to focus on your total feeling of exertion.

| 0 | Nothing at all |
| --- | --- |
| 0.5 | Very, very light |
| 1 | Very light |
| 2 | Light |
| 3 | Moderate |
| 4 | Somewhat hard |
| 5 | Hard |
| 6 |  |
| 7 | Very hard |
| 8 |  |
| 9 |  |
| 10 | Very, very hard |

*Note:* Borg GA. Psychophysical bases of perceived exertion. *Med Sci Sports Exerc*. (1982) 14(5):377*–*81.
